# Year-round efficacy of a single treatment of fluralaner injectable suspension (Bravecto Quantum™) against repeated infestations with *Rhipicephalus sanguineus* (sensu lato) and* Ctenocephalides felis* in dogs

**DOI:** 10.1186/s13071-023-05960-5

**Published:** 2023-10-23

**Authors:** Petr Fisara, Frank Guerino

**Affiliations:** 1 Animal Health, Macquarie Park, NSW 2113 Australia; 2 Merck Animal Health, 126 E. Lincoln Avenue, Rahway, NJ 07065 USA; 3 Merck Animal Health, 126 E. Lincoln Avenue, Rahway, NJ 07940 USA

**Keywords:** Bravecto, *Ctenocephalides felis*, Flea, Fluralaner, Injectable, *Rhipicephalus sanguineus *(sensu lato), Tick

## Abstract

**Background:**

Poor owner compliance with monthly control measures means that dogs in Australia can remain susceptible to infestations with fleas, present throughout the whole year, and brown dog ticks, which thrive in tropical and subtropical areas. A 150 mg/ml injectable fluralaner suspension (Bravecto Quantum™) was developed to help ensure year-round protection against these parasites. A study investigated the persistent efficacy of this formulation against repeated challenges with *Rhipicephalus sanguineus *(sensu lato) and *Ctenocephalides felis* throughout 12 months following a single subcutaneous treatment.

**Methods:**

Twenty dogs were blocked by pre-treatment *R. sanguineus *s.l. counts and randomized to an untreated control group or to a group treated once, on day 0, with the fluralaner injection (15 mg/kg). Infestations of 50 mixed-sex, unfed adult *R. sanguineus *s.l. and up to 100 *C. felis* were done on days 7, 14, 35, 63, 91, 126, 154, 182, 210, 245, 273, 301, 336 and 365. Live flea and tick counts were completed 48 h post-infestation. Flea infestations were also done on day −1, with counts on day 2. Infestations were considered adequate if at each evaluation, at least six dogs in the control group retained at least 20% of tick challenges and 25% of flea challenges.

**Results:**

The fluralaner injectable suspension was well tolerated. Efficacy against existing flea infestations was > 99% (arithmetic and geometric means) at 48 h post-treatment. At all subsequent assessments throughout the year following treatment, efficacy against fleas remained at 100%. Arithmetic mean tick count reductions relative to the control group ranged from 97.6% to 100% from day 7 through 11 months and was 92.6% at 12 months (geometric means 95.2% to 100% through 12 months).

**Conclusion:**

The injectable fluralaner suspension was effective against fleas and brown dog ticks for 12 months following a single treatment. Compared with more frequently administered products where compliance may be suboptimal, the year-round efficacy of this veterinarian-administered fluralaner formulation has the potential to improve owner compliance for control of fleas and ticks. In turn, by reducing the detrimental effects of flea and tick infestations and risk of transmission of flea- and tick-borne pathogens, canine health can be enhanced.

**Graphical Abstract:**

**Figure:**
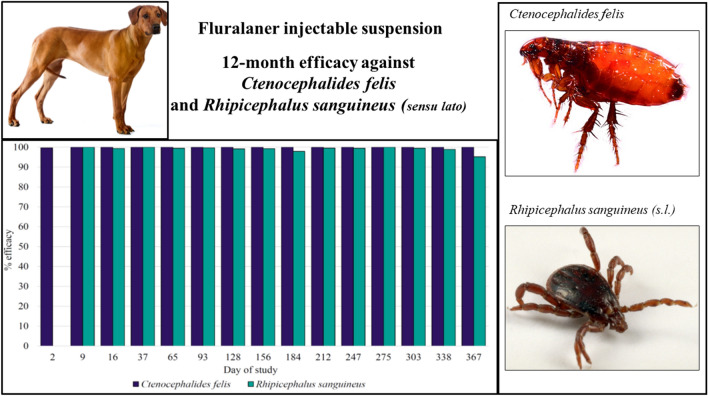

## Background

The cat flea *Ctenocephalides felis* is the most common flea species parasitizing cats and dogs [1], and due to favourable climatic conditions, dogs in Australia are frequently infested throughout the whole year. Within minutes of finding a host, fleas begin feeding on blood, and start producing eggs within 24 to 48 h [1]. An adult female flea can produce 40 to 50 eggs per day, which fall from the host and develop into adults in the environment within several weeks [1]. Besides causing discomfort to animals and humans through biting, fleas cause flea allergy dermatitis (FAD) [2] and are vectors for bacteria, such as *Bartonella* and *Rickettsia* species, which can cause disease in both humans and animals [3–5]. Fleas are also an intermediate host for the tapeworm *Dipylidium caninum*, which can be transferred to humans, especially children and infants [6–8]. Therefore, flea control is important not only for animal health but also for human health, due to the potential for transmitting pathogens that present a zoonotic risk.

The brown dog tick, *Rhipicephalus sanguineus* (sensu lato), is an important parasite to the medical and veterinary communities due to vector competence for several pathogens. Dogs are the primary host of this tick, which thrives in tropical and subtropical areas where its host-seeking aggressiveness is enhanced by a warm climate [9–11]. The expanding geographic distribution of *R. sanguineus *s.l. brings with it increased risk of transmission of pathogens to its canine and sometimes human hosts [12]. Of particular concern, in 2020, *Ehrlichia canis*, the causative agent of canine monocytic ehrlichiosis, was confirmed for the first time in Australian dogs in the Kimberley region of Western Australia and in the Northern Territory [13]. Since that time, infections detected in northern South Australia and north-west Queensland indicate that the tick-borne spread of this pathogen continues [13].

The prevalence and associated risks arising from infestation with fleas and brown dog ticks drives an ongoing need for innovative and effective flea and tick control measures. While a recent survey found that most veterinarians in Australia recommend year-round prophylaxis against fleas and ticks [14], achieving that level of protection is complicated by poor pet-owner compliance with the required frequency of treatments [15]. As an orally administered formulation for dogs, the long-acting isoxazoline compound fluralaner is labelled in Australia to provide 3-month efficacy against fleas, 4-month efficacy against *Ixodes holocyclus*, and 2-month efficacy against *R. sanguineus *s.l. [16].

A report showing that dog owners administering fluralaner were more compliant with veterinary recommendations, compared with recommendations requiring monthly administration, concluded that a longer duration of efficacy means reduced risk of missed treatments that leave dogs susceptible to infestations [17]. Year-round prophylaxis is regarded as the gold standard for control of ectoparasites [18].

As reduced need for frequent treatments can improve compliance, an injectable formulation of fluralaner has been designed to provide a full year of protection following a single administration. A recent laboratory study demonstrated that this novel fluralaner injectable suspension provided efficacy > 95% for at least 13 months against canine infestations with *I. holocyclus* [19]. To further evaluate the effectiveness of this fluralaner formulation, this study investigated the duration of protection provided against infestations with *C. felis* and *R. sanguineus* s.l.

## Methods

This was a dose confirmation, non-masked, randomized complete block design study, with a fluralaner-treated and an untreated control group. The objective was to investigate therapeutic efficacy against *C. felis* and persistent efficacy against challenges of dogs with *R. sanguineus *s.l. and *C. felis* for 12 months following a single subcutaneous treatment with fluralaner 150 mg/ml injectable suspension. The study protocol was designed to conform to the VICH GCP guidelines [International Co-operation on Harmonisation of Technical Requirements for Registration of Veterinary Medicinal Products, Good Clinical Practice (GL9)], June 2000 [20]; the World Association for the Advancement of Veterinary Parasitology (WAAVP) second edition: guidelines for evaluating the efficacy of parasiticides for the treatment, prevention and control of flea and tick infestation on dogs and cats [21]; and the Australian Pesticides and Veterinary Medicines Authority (APVMA) Preamble for the WAAVP guideline for fleas and ticks on dogs and cats [22]. 

### Dogs and management

For inclusion in the study, dogs were required to be clinically healthy, at least 6 months of age and free from any residual acaricidal efficacy, as demonstrated by flea and tick carrying capacity determined by infestations and assessments in the week prior to treatment. None of the dogs had received any acaricidal treatment in the 12 months prior to the study. Any dog that was debilitated, suffering from disease or injury, pregnant or lactating, fractious or otherwise unsuitable was excluded. The occurrence of flea allergy dermatitis was closely monitored before and during the study.

On non-infestation days, dogs were housed in socially compatible pairs or groups of three, within the treatment group, in adjacent concrete-floored pens of solid fibre-reinforced plastic panels with mesh above, and each pen was provided with a raised resting platform. Feed was provided to meet the nutrient and energy requirements for ensuring the well-being of the dogs. Water was from a single source and was available ad libitum via automatic troughs. Routine prophylactic concurrent medications, including anthelmintics (excluding products containing macrocyclic lactones), were continued during the study, but not within 14 days of day 0, and not coinciding with critical study activities such as flea and tick infestations. Medical conditions were treated as needed following a veterinary examination. On non-infestation days, and weather permitting, compatible dogs were exercised within treatment groups for at least 30 min per day in designated outdoor grassed runs of approximately 500 m^2^. There was no contact possible between dogs of different treatment groups, which were separated by an exercise yard containing non-study dogs. Dogs were monitored by a qualified individual three times daily from the start of the acclimatization on day −14 until study completion on day 367.

During infestation cycles, elevated cages were set up within pens to avoid environmental contamination by escaped ticks and to prevent endemic ticks from infesting study animals, potentially confounding study results. The cages provided floor space of ~2 m^2^ and stood on ~10 cm stilts placed in a bath filled with water and detergent. Two aluminium trays, also filled with water and detergent, were placed under each elevated cage.

### Randomization and treatment

From 24 uniquely identified dogs, the 20 with the highest live tick counts on day −5, 48 h post-infestation, were selected for the study. All selected dogs were beagles aged between 2.1 and 8.3 years. The animals weighed 12.7 to 23.3 kg on day −1, 14 were male (one intact) and six were female (four had been spayed). Based on the day −5 tick counts, dogs were ranked and then sequentially blocked into pairs (in the control and treated groups, mean day −5 flea counts were 47.3 and 39.3, respectively, and 12.1 and 12.4 for ticks). Within each block, dogs were randomly allocated to a treatment group using the *randbetween* function in Microsoft Excel, resulting in 10 dogs in each group. Dogs in the control group remained untreated, whilst dogs allocated to the fluralaner treatment group received a single subcutaneous injection of the fluralaner suspension (150 mg/ml), administered on a single occasion (day 0) in the dorsoscapular region. The dogs were treated at the fluralaner label dose rate of 15 mg/kg (0.1 ml/kg), calculated according to body weight recorded on day −1. Clinical observations of fluralaner-treated dogs were conducted over the 5 min immediately following treatment administration. Injection site assessments included visual assessments and gentle palpation for swelling, erythema, heat and pain. These assessments were conducted on all dogs prior to treatment and at 24, 48, 72 and 96 h post-treatment, and then on days 7, 10 and 14. Clinical observations commenced on all dogs at 1 h, 2 h (±15 m) and 24 h (±30 m) following treatment of the last dog.

### Infestations and assessments

The *R. sanguineus *s.l. strain used in the study originated from wild-caught ticks collected from various locations in Darwin, Northern Territory, and in North Queensland for an efficacy study in 2014. Following the study, the ticks became endemic in the kennels. Since November 2019, this strain has been maintained on dogs at the facility and had never been tested for tolerance to ectoparasiticides. The sex ratio of ticks used for infestations was checked on one occasion, from a subsample of 192 ticks, and found to be 58% female and 42% male. Fleas were of the Scarborough *C. felis* strain, originally collected in 2015 in Brisbane, Australia, from a household with a history of fipronil/*S*-methoprene treatment failure. The flea colony was maintained off-site, and flea pupae were supplied during the week prior to each infestation. The flea population had not been pressured by any insecticidal treatments and had one infusion of a New South Wales field strain collected in May 2020 from untreated household dogs. An in vitro bioassay in June 2015 confirmed that the strain carried tolerance to fipronil. Regular retesting, most recently in June 2021, confirmed that the status of tolerance to fipronil had remained in the colony.

Each dog was infested with 50 unfed mixed-sex adult *R. sanguineus *s.l. The animals were restrained but not sedated for the procedure. The ticks were released onto body locations such as the head, neck and dorsum to simulate the tick’s natural predilection for these areas. Immediately after tick infestation, 100 unfed adult fleas were released from a vial onto the dorsum of each dog. On two occasions, at 35 days and 63 days post-treatment, the dogs were infested with less than 100 fleas due to the slower-than-expected hatching of adult fleas from pupae. Following the release of the ticks and fleas, dogs were restrained for one more minute and then given a bone or other suitable item, such as a dental stick, to distract them and minimize the likelihood of the parasites being groomed off immediately following infestation.

To screen dogs for study suitability, flea and tick infestations were completed on day −7 and counts were done on day −5; however, only tick counts were used for allocations. For efficacy assessments, flea infestations were completed on day −1 and flea and tick infestations were completed on days 7, 14, 35, 63, 91, 126, 154, 182, 210, 245, 273, 301, 336 and 365.

Counts of live fleas were performed 48 (±4) h post-treatment to determine efficacy against existing infestations. Counts of live fleas and ticks were performed 48 h after each subsequent infestation to determine persistent efficacy. Counts were performed in two stages: a finger and visual search followed by thorough combing using flea combs. The finger and visual search began with the areas on which ticks had been released and was followed by a whole-body search to locate any ticks that may have migrated from the release sites. The 10-min combing period then removed live fleas and any remaining ticks. Ticks were removed by fingers or forceps [21]. Study dogs were well accustomed to the procedure, and neither sedation nor restraint was needed. Whenever a flea or tick was found within the last minute of combing, the combing continued for another full minute until no fleas or ticks were recovered within a minute of combing. Live flea and tick counts and assessments were performed by teams of two to three individuals. Ticks were classified as live (attached or free) or dead (attached or free) [21]. If a tick appeared normal (i.e., not dried out) but was immobile, it was stimulated by exposure to carbon dioxide by breathing on the tick and manually touching it with a probe. Any tick demonstrating the slightest movement, however feeble, was classified as live. Any tick demonstrating no movement and no reaction was classified as dead.

Local skin reactions to irritation caused by the parasite infestations were treated with a topical local anaesthetic, corticosteroid and antibiotic. Mild tick attachment reactions (inflammation, alopecia, redness with serum oozing) were expected during the study and, when mild, could be treated by applying a dab of topical iodine solution with a cotton wool ball or gauze swab following tick removal.

### Analysis

A minimum of six animals per group is recommended for laboratory-based dose determination and confirmation studies of insecticides and acaricides for use in dogs and cats [21]. Increasing the number of study animals can improve the reliability of results, so 10 dogs were included in each study group. Infestations were considered adequate if at each evaluation, at least 20% of the tick challenges and 25% of the flea challenges [21] were found on at least six dogs in the untreated control group.

The experimental unit was the individual animal. The individual live flea and tick counts from each dog were used to calculate the arithmetic and geometric means for each group on each count day. The count data were transformed prior to analysis using the *y* = log_e_(*x* + 1) transformation. The transformed counts were analysed using a linear model. Separate analyses were conducted at each time point. Fixed-effect-only models were used in the analysis. At each time point, a two-sided *t*-test was used to assess whether the difference in least-squares means between the control and fluralaner-treated groups was statistically significant at *P* ≤ 0.05. These comparisons were performed on the transformed scale. The least-squares mean counts were then back-transformed to obtain the mean counts on the response scale. Data for live tick counts and live flea counts were summarized as arithmetic and geometric means by treatment group and time point. Efficacy was estimated as the reduction in arithmetic and geometric means, respectively, in the treated group compared with the control group, using the formula:

$$\% \mathrm{reduction}=100 \times \frac{\mathrm{mean\, count\, control\, group}-\mathrm{mean\, count\, treated\, group}}{\mathrm{mean\, count\, control\, group}}$$

The software R version 4.0.5 was used for the analysis [23].

## Results and discussion

The minimum requirement for adequacy of infestation of the untreated control group was maintained at all flea and tick assessment time points (at 48-h counts, at least 20% and 25% of the tick and flea infestation dose, respectively, was retained on at least six dogs). On two challenge days, the low numbers of available fleas meant that the infestation dose of both groups had to be reduced. Thus, on day 35, half the dogs in each group received 50 fleas, while the other half were challenged with 100 fleas. Similarly, on day 63, half the dogs in each group were challenged with 75 fleas while half were challenged with 100 fleas. The criteria for adequate infestations were met on both count days despite the differences in the number of fleas used for infestations. On day 185, three untreated control dogs required treatment for mild flea bite reactions.

Injection site assessments were performed on all dogs prior to treatment on day 0 and then on days 1, 2, 3, 4, 7, 10 and 14. Mild injection-site erythema observed in one dog at 24 h post-treatment had resolved spontaneously by the observation on the next day. No other dogs experienced skin reactions, and there were no treatment-related adverse events.

**Fig. 1:**
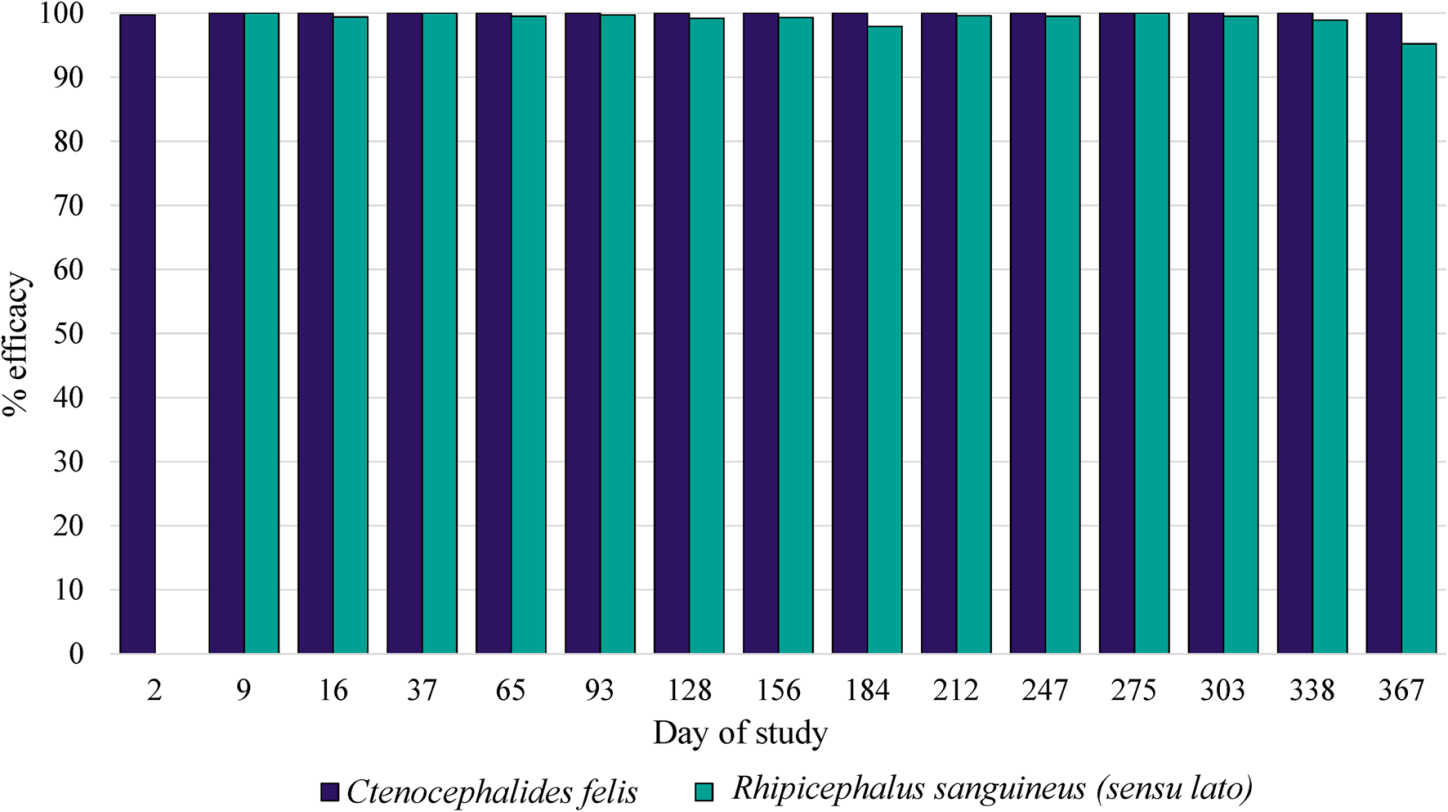
Mean (geometric) efficacy of a single administration on day 0 of the fluralaner injectable suspension (15 mg/kg) against experimental infestations with *Ctenocephalides felis* and *Rhipicephalus sanguineus* s.l.

In the untreated control group, arithmetic mean live flea counts ranged from 43.8 on day 247 to 75.9 on day 2 and day 16 (Table 1). In the fluralaner-treated group, compared with the control group, efficacy against existing flea infestations (day 2 counts) was 99.7% and 99.8% based on arithmetic and geometric means, respectively (Fig. 1). At each subsequent assessment throughout the year following treatment, efficacy against fleas was sustained at 100%.

**Table 1:** Arithmetic (geometric) mean counts of live *Ctenocephalides felis* and percentage reductions after each infestation following a single treatment of dogs with fluralaner subcutaneous injection (15 mg/kg) on day 0

Days after treatment	Mean flea counts arithmetic (geometric)		% Efficacy, arithmetic (geometric) mean	Statistics	
	Control	Fluralaner		*t*-value	*P*-value
2	75.9 (67.8)	0.2 (0.1)	99.7 (99.8)	*t*_(16)_ = 6.4	< 0.001
9	59.7 (57.4)	0.0 (0.0)	100.0 (100.0)	*t*_(16)_ = 16.7	< 0.001
16	75.9 (72.7)	0.0 (0.0)	100.0 (100.0)	*t*_(16)_ = 15.1	< 0.001
37^a^	45.2 (41.9)	0.0 (0.0)	100.0 (100.0)	*t*_(16)_ = 13.2	< 0.001
65^b^	46.6 (41.5)	0.0 (0.0)	100.0 (100.0)	*t*_(16)_ = 8.9	< 0.001
93	58.4 (52.8)	0.0 (0.0)	100.0 (100.0)	*t*_(16)_ = 8.5	< 0.001
128	56.1 (50.05)	0.0 (0.0)	100.0 (100.0)	*t*_(16)_ = 7.6	< 0.001
156	54.5 (51.8)	0.0 (0.0)	100.0 (100.0)	*t*_(16)_ = 12.6	< 0.001
184	45.5 (34.3)	0.0 (0.0)	100.0 (100.0)	*t*_(16)_ = 5.1	< 0.001
212	49.3 (45.7)	0.0 (0.0)	100.0 (100.0)	*t*_(16)_ = 11.2	< 0.001
247	43.8 (41.5)	0.0 (0.0)	100.0 (100.0)	*t*_(16)_ = 12.2	< 0.001
275	49.7 (45.4)	0.0 (0.0)	100.0 (100.0)	*t*_(16)_ = 9.7	< 0.001
303	44.2 (38.6)	0.0 (0.0)	100.0 (100.0)	*t*_(16)_ = 7.9	< 0.001
338	55.1 (48.19)	0.0 (0.0)	100.0 (100.0)	*t*_(16)_ = 7.0	< 0.001
367	47.9 (43.2)	0.0 (0.0)	100.0 (100.0)	*t*_(16)_ = 8.3	< 0.001

Targeted challenge on each day was 100 fleas per dog

^a^Due to a shortage of fleas, 50% of the dogs in each group were infested with 50 fleas

^b^Due to a shortage of fleas, 50% of the dogs in each group were infested with 75 fleas

For *R. sanguineus *s.l., arithmetic mean live tick counts from the control group ranged from 11.8 on day 16 to 23.6 on day 93 (Table 2). Efficacy of the fluralaner injectable formulation was 100% on day 9, and arithmetic and geometric mean tick count reductions relative to the control group remained ≥ 97.6% until day 338. On day 367, the arithmetic and geometric mean tick count reductions were 92.6% and 95.2%, respectively. Statistically significant differences were detected in mean flea (*t*_(16)_ = 5.1–16.7, *P* < 0.001) and tick (*t*_(16)_ = 6.9–14.1, *P* < 0.001) counts between the groups at all post-treatment assessments.

**Table 2:** Arithmetic (geometric) mean counts of live *Rhipicephalus sanguineus* (sensu lato) and percentage reductions after each infestation following a single treatment of dogs with fluralaner subcutaneous injection (15 mg/kg) on day 0

Days after treatment	Mean tick counts arithmetic (geometric)		% efficacy, arithmetic (geometric) mean	Statistics	
	Control	Fluralaner		*t*-value	*P*-value
9	12.0 (12.0)	0.0 (0.0)	100.0 (100.0)	*t*_(16)_ = 45.1	< 0.001
16	11.8 (11.3)	0.1 (0.1)	99.2 (99.4)	*t*_(16)_ = 10.3	< 0.001
37	18.9 (18.2)	0.0 (0.0)	100.0 (100.0)	*t*_(16)_ = 14.2	< 0.001
65	14.2 (13.2)	0.1 (0.1)	99.3 (99.5)	*t*_(16)_ = 8.7	< 0.001
93	23.6 (22.7)	0.1 (0.1)	99.6 (99.7)	*t*_(16)_ = 14.1	< 0.001
128	18.7 (17.8)	0.2 (0.1)	98.9 (99.2)	*t*_(16)_ = 9.3	< 0.001
156	12.2 (10.5)	0.1 (0.1)	99.2 (99.3)	*t*_(16)_ = 6.7	< 0.001
184	16.6 (15.5)	0.4 (0.3)	97.6 (97.9)	*t*_(16)_ = 7.8	< 0.001
212	19.4 (17.0)	0.1 (0.1)	99.5 (99.6)	*t*_(16)_ = 6.9	< 0.001
247	14.7 (13.2)	0.1 (0.1)	99.3 (99.5)	*t*_(16)_ = 8.4	< 0.001
275	21.7 (19.1)	0.0 (0.0)	100.0 (100.0)	*t*_(16)_ = 7.8	< 0.001
303	17.0 (15.1)	0.1 (0.1)	99.4 (99.5)	*t*_(16)_ = 10.3	< 0.001
338	18.0 (17.1)	0.3 (0.2)	98.3 (98.9)	*t*_(16)_ = 8.1	< 0.001
367	13.5 (11.6)	1.0 (0.6)	92.6 (95.2)	*t*_(16)_ = 4.7	< 0.001

Dogs were infested with 50 brown dog ticks at all infestations

The year-long efficacy shown in this study is consistent with the duration of efficacy shown against the paralysis tick [19], making the fluralaner suspension the first injectable ectoparasiticide to offer 12-month control of fleas and ticks on dogs with a single treatment. Importantly, by killing fleas before they can lay eggs, fluralaner prevents new fleas from establishing an infestation in a household throughout the 12 months following treatment [24]. In households in which flea pupae and larvae are already present, for the year following fluralaner treatment, any newly emerging fleas will be killed before egg-laying begins. As pre-emergent fleas can remain viable for longer than 6 months [1], the 12-month efficacy duration of this fluralaner formulation will provide a progressive depletion of the household flea population.

Household infestations with brown dog ticks are common in many parts of the world, as this tick is well adapted to living within human dwellings as well as in gardens and kennels when hosts are present [25]. Thus, the prolonged efficacy of this fluralaner injectable formulation can provide reliable long-lasting protection against brown dog tick populations on dogs and help to reduce infestations that may have established in households.

## Conclusion

The injectable fluralaner suspension was effective against fleas and brown dog ticks for 12 months following a single treatment. Compared with more frequently administered products, the extended efficacy shown by this veterinarian-administered fluralaner injection has the potential to improve owner compliance with recommendations for control of fleas and ticks. In turn, reliable control of fleas and ticks can enhance canine health and help reduce the risk of transmission of flea- and tick-borne pathogens.

## Data Availability

Data from this study are proprietary and maintained by Merck Animal Health, Rahway, NJ, USA.

## Abbreviations

APVMA: Australian Pesticides and Veterinary Medicines Authority
GCP: Good Clinical Practice
VICH: International Co-operation on Harmonisation of Technical Requirements for Registration of Veterinary Medicinal Products
WAAVP: World Association for the Advancement of Veterinary Parasitology
